# Accelerated global sensitivity analysis of genome-wide constraint-based metabolic models

**DOI:** 10.1186/s12859-021-04002-0

**Published:** 2021-04-26

**Authors:** Marco S. Nobile, Vasco Coelho, Dario Pescini, Chiara Damiani

**Affiliations:** 1 Department of Informatics, Systems and Communication, University of Milano-Bicocca, Milan, Italy; 2 Department of Statistics and Quantiative Methods, University of Milano-Bicocca, Milan, Italy; 3 Department of Biotechnology and Biosciences, University of Milano-Bicocca, Milan, Italy; 4 SYSBIO/ISBE.IT Centre for Systems Biology, Milan, Italy; 5 Department of Industrial Engineering and Innovation Sciences, Eindhoven University of Technology, Eindhoven, The Netherlands

**Keywords:** Global sensitivity analysis, Sobol coefficients, Genome-wide models, Flux Balance Analysis, High-performance computing

## Abstract

**Background:**

Genome-wide reconstructions of metabolism opened the way to thorough investigations of cell metabolism for health care and industrial purposes. However, the predictions offered by Flux Balance Analysis (FBA) can be strongly affected by the choice of flux boundaries, with particular regard to the flux of reactions that sink nutrients into the system. To mitigate possible errors introduced by a poor selection of such boundaries, a rational approach suggests to focus the modeling efforts on the pivotal ones.

**Methods:**

In this work, we present a methodology for the automatic identification of the key fluxes in genome-wide constraint-based models, by means of variance-based sensitivity analysis. The goal is to identify the parameters for which a small perturbation entails a large variation of the model outcomes, also referred to as sensitive parameters. Due to the high number of FBA simulations that are necessary to assess sensitivity coefficients on genome-wide models, our method exploits a master-slave methodology that distributes the computation on massively multi-core architectures. We performed the following steps: (1) we determined the putative parameterizations of the genome-wide metabolic constraint-based model, using Saltelli’s method; (2) we applied FBA to each parameterized model, distributing the massive amount of calculations over multiple nodes by means of MPI; (3) we then recollected and exploited the results of all FBA runs to assess a global sensitivity analysis.

**Results:**

We show a proof-of-concept of our approach on latest genome-wide reconstructions of human metabolism Recon2.2 and Recon3D. We report that most sensitive parameters are mainly associated with the intake of essential amino acids in Recon2.2, whereas in Recon 3D they are associated largely with phospholipids. We also illustrate that in most cases there is a significant contribution of higher order effects.

**Conclusion:**

Our results indicate that interaction effects between different model parameters exist, which should be taken into account especially at the stage of calibration of genome-wide models, supporting the importance of a global strategy of sensitivity analysis.

**Supplementary Information:**

The online version contains supplementary material available at 10.1186/s12859-021-04002-0.

## Background

Detailed computational models of metabolism are increasingly being reconstructed and simulated for many organisms, ranging from prokaryotes to *Homo sapiens*, with the aim of connecting genotype with metabolic phenotype [1]. They have extensively being applied within metabolic engineering, for instance to optimize the cells’ production of a certain substance, and hold great potential in unraveling the fragility points of complex pathological diseases in which a rearrangement of metabolism plays an essential role [2] (e.g., cancer, diabetes, or neurodegenerative disorders).

These genome-wide metabolic networks encompass all the reactions that can be catalyzed by the enzymes that are encoded in a given genome. In the case of human metabolism, they include more than 10.000 biochemical reactions [3]. Notwithstanding the advancements in dynamic simulation [4, 5], using reaction-based or hybrid approaches [6], the analysis of large-scale biochemical models can still be challenging because some mandatory information (e.g., kinetic parameters of rate laws, the amounts of chemical species) is still largely undetermined [7]. For this reason, these networks are typically investigated by means of constraint-based models (CBMs) [8], and in particular of Flux Balance Analysis [1] (FBA).

Although CBMs are not fit for the analysis of molecular networks in general, they are well suited for metabolic networks, as the concentration of intracellular metabolites in time can be reasonably approximated to a constant value. Despite neglecting information on transient dynamics, CBMs represent a means for the identification of key features of metabolism such as growth yield, network robustness, and gene essentiality. For instance, in the case of unicellular organisms, steady-state extracellular fluxes (e.g., consumption rate of carbon and nitrogen) could be derived from chemostat experiments. Relying on these constraints and upon fitting of two maintenance energy parameters (growth and non growth associated maintenance) FBA often correctly predicted the expected relative growth yields [9]. In the case of human CBMs, it is far more difficult to identify proper constraints for FBA, especially when the aim is the investigation of metabolic behavior of human cells in vivo. It is indeed impracticable to estimate the value of each extracellular flux, considering that the latest curated version of human metabolic network Recon 3D [3] takes into account 1559 nutrients that can be exchanged with the environment. On the other hand, if the influx of all these metabolites is left unbound, the resulting phenotype might be very different from biological reality. Constraining a limited subset of them may lead to even worse predictions [10]. Hence, assessing the relative influence of these boundaries on the model outputs is fundamental, not only to identify the extracellular fluxes that should be tightly constrained, but also to determine which inputs are most correlated with the output of the system. In the specific case of cancer metabolism, this could help to investigate which nutrients affect cancer growth, providing insights about novel treatments.

To this aim, we have previously proposed to perform sensitivity analysis (SA) to rank model boundaries according to their contribution to model dynamics [11]. The goal of SA is to investigate how the uncertainty in the output of a mathematical model can be divided and allocated to different sources of uncertainty in its inputs. Specifically, SA methods consist in computing a sensitivity coefficient for each model parameter and are typically classified into local and global approaches. This classification of SA approaches emphasizes how the input parameters space of the model is explored: exploiting a local variation around a starting base point, in the former case; extensively exploring the input factors, in the latter case. Local methods are more established, computationally less intensive than global ones and most informative in linear systems where local properties can be easily generalized to other space regions, but they require a baseline parametrization. Because baseline values of extracellular fluxes should be set according to experimental measures and this information is often not available, global methods must thus be applied. In particular, variance-based methods calculate how much of the variance of an output values is explained by a given input and allow to asses the effect of interactions among inputs [12].

We have preliminary explored the application of variance-based global methods on small metabolic models [11]. Nevertheless, when dealing with genome-wide models, sequential simulations for the computation of sensitivity indices become impracticable. By way of examples, a global SA of the latest genome wide human reconstruction Recon 3D [3] would require the perturbation of 1559 exchange reactions and approximately 12.7 million FBA optimizations for a reasonable sampling of the parameter space. On a workstation equipped with a 9th Gen Intel CPU, the analysis would therefore take several weeks to be completed and about 100 GB of RAM.

**Fig. 1:**
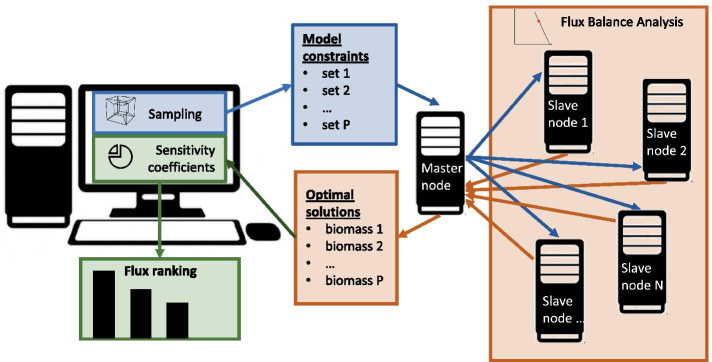
Workflow of distributed computation of global sensitivity coefficients and ranking of constraint-based model parameters

In this work, we propose a workflow that can be efficiently applied to any genome-wide constraint-based model. To compute partial and total sensitivity coefficients, we perform the Sobol’s method for variance-based sensitivity analysis [12, 13], combined with the random sampling scheme of parameters proposed by the Saltelli et al. [14]. Saltelli’s method generates quasi-random sequences by extending Sobol’s method, to the aim of reducing the error rates in the resulting sensitivity index calculations [13]. To accelerate this computationally challenging process, we take advantage of the mutual independence between FBA optimizations, by distributing the optimizations on multiple cores. An overview of the approach is depicted in Fig. 1.

As a proof of principle, we show an application of the approach to latest human metabolic reconstructions Recon2.2 [15] and Recon3D [3]. We perform a sensitivity analysis on all exchange reactions included in the genome-wide models. We also report SA results for a limited set of exchange reactions that are expected to influence the optimal growth rate [16].

## Materials and methods

### Constraint-based modeling

Constraint-based modeling lays its foundations on the mass balance equation $S \vec{v} = \vec{0}$, where $\vec{v}$ is the vector of flux values of each network reaction, whereas *S* is a $m \times r$ sparce matrix that reports the stoichiometric coefficients of each of the *m* network metabolites in each of the *r* network reactions. Metabolites that are not either substrate or products of a given reactions have a null coefficient. The kernel of the stoichioemetric matrix *S* describes all the possible states whose image is null, thus representing the accessible steady states of the system. This kernel, together with the steady state assumption allows to identify the bounded solution space of all feasible flux distributions in an under-determined system, a common characteristic of metabolic networks which usually have more fluxes than metabolites ($r > m$). In order to closer mimic the organism behaviour in a given experimental set-up, it is possible to add several constraints such as the direction or the capacity associated to each reaction by specifying the maximum and minimum values of the fluxes. Given the assumption that it is possible to translate the organism innate tendency to accomplish a specific function into a specific mathematical objective function, FBA [17] allows to determine an optimal flux distribution for the model to satisfy the given aim. In the framework so far presented, the description of the organism metabolic processes in a linear system fashion allows to exploit Linear Programming to obtain the optimal flux distribution of interest. It is thus possible to postulate the linear programming problem as the maximization of the scalar product $\vec{w} \cdot \vec{v}$ subject to the constraints $S \vec{v} = 0$ and $\vec{v}_{l} \le \vec{v} \le \vec{v}_{u}$, with $\vec {w}$ being the objective coefficients vector, $\vec{v}_{l}$ and $\vec{v}_{u}$ the lower and upper bounds allowed for the model fluxes. Linear programming methods are not computationally demanding, a key characteristics to pave the way to the analysis of metabolism at the genome-wide scale.

### Sensitivity analysis

SA is a computational methodology designed to investigate how the uncertainty in the output of a given mathematical model can be caused by different sources of uncertainty in its inputs [18]. The outcome of a SA run is typically a sorted list of the sensitivity coefficients associated to the aforementioned inputs [19, 20]. Several SA methods exist for the analysis of biological models (notably, Morris’ elementary-effects [21, 22], variance-based sensitivity [12, 14], and derivative-based sensitivity [23]).

The goal of this work is to determine how the uncertainty of flux boundaries on a FBA model affects its objective function; in this paper, we considered the objective of biomass maximization. It is worth noting that our methodology is absolutely general and can be applied to any alternative objective function.

More specifically, we aimed at determining how intake fluxes, formalized as a set of *k* parameters, affect the growth of an organism. We quantified the uncertainty by bounding each parameter in a reasonable interval. In this work, we considered the scenario in which the reference value of each parameter is not known. It is common practice in genome-wide modeling to bound nutrient influxes to a value which is sufficiently low as compared to the boundary of internal reactions - in order to avoid internal boundaries to become limiting - and sufficiently high to cover the stoichiometric coefficients of reaction involving them. Conventionally, intake fluxes have negative sign (flux in the backward direction). In light of the above considerations, we partially arbitrarily set the boundaries of exchange in the interval [-10, 0] (mM/h), with the intent of globally sampling virtually all possible ratios between all intake fluxes. In fact, when setting constraints on flux boundaries, the relative values of boundaries, more than their absolute value, determine the optimal flux distribution.

The sensitivity analysis focused on the impact of the perturbation of the parameter corresponding to the lowest boundary. Hence, we performed a variance decomposition based global sensitivity analysis in this interval, varying all parameters simultaneously instead of a one-at-a-time policy.

We performed the SA by means of Sobol’s variance-based method [12], using the implementation provided by the SAlib library [24]. Given *D* variables in the model, the method determines the sensitivity indices by relying on $N\; ( 2\; D + 2)$ independent random samples of the parameters space, where *N* is a user-defined parameter. Such samples are obtained with the Saltelli approach [14].

We computed two different kinds of information: the first order index $S_i$ (Eq. 1), which represents the main effect contribution of the input factor $X_i$ to the variance of the output *Y*; the total effect index $S_{T_i}$ (Eq. 2), which accounts for the total contribution to the output variation due to each factor, i.e, its first-order effect plus all higher-order effects due to interactions [25].

1 $$\begin{aligned} S_{i}&= \frac{Var[E(Y|X_{i})]}{Var(Y)} \end{aligned}$$

2 $$\begin{aligned} S_{T_i}&= \frac{E[Var(Y|X_{\sim i})]}{Var(Y)} \\ &= 1 - \frac{Var[E(Y|X_{ \sim i})]}{Var(Y)} \end{aligned}$$

with $Y|x_{i}$ being the conditioning over each factor *i*, whereas $Y|x_{\sim i}$ is the conditioning over all factors but *i*.

To estimate the variability of the above indices and obtain confidence intervals, we used bootstrap methods, as described in [26]. Basically, the $N\; ( 2\; D + 2)$ sampled parametrizations were resampled (i.e., sampled with replacement) 100 times for each variable and the indices were recalculated, leading to a bootstrap estimate of the sampling distribution of the sensitivity indices.

Once the variables are determined, along with the intervals of variation of each input variable, a sensitivity analysis can be decomposed into three separate phases:

1. sample the model’s parameterizations to be tested;
2. evaluate the output of the model’s for each parameterization;
3. collect all the outputs and calculate the sensitivity coefficients.

It is worth noting that steps 1 and 3 are inherently atomic, while the step 2 can be performed in parallel due to the fact that all evaluations are based on separate and independent FBA runs.

### High performance computing

Although SA represents a powerful tool to investigate the behavior of a model, in the case of genome-wide systems it can become computationally challenging: as a matter of fact, a proper investigation of the multi-dimensional boundaries space leads to a combinatorial explosion of configurations to be tested and optimized. However, all linear programming optimizations are mutually independent, hence it is possible to mitigate the exceptional computational effort by offloading the calculations to a parallel, or distributed, architecture.

Message Passing Interface (MPI) is a communication protocol for parallel computing in multi-core and multi-node architectures. MPI is the *de facto* standard for intra- and inter-node information exchange in distributed-memory computing clusters executing parallel code. OpenMPI is the most widespread open-source implementation of MPI, available in basically every super-computer. The mpi4py package [27] provides bindings of the the MPI standard for the Python programming language, allowing programs to exploit systems with multiple processors by means of an object-oriented “pytonish” interface.

In this work, we exploited MPI to distribute the FBA optimizations over the computing nodes of the D.A.V.I.D.E. (Development of an Added Value Infrastructure Designed in Europe), an extremely performing (peak power: 1 petaFLOP) and energy-efficient super-computer realized by the italian consortium for super-computing CINECA. D.A.V.I.D.E. is composed of 45 nodes, equipped with 2 POWER8 CPUs and interconnected with 100 GB/s InfiniBand links. In our master-slave paradigm, one computing node (the master) was responsible for generating the parameterizations to be tested with FBA and distributing the calculations to the other (slave) nodes. As soon as a slave completes its calculations, it asynchronously communicates the result to the master using a MPI message. When all results are collected, the master computes the sensitivity indices, creates the ranking and returns the result to the user (see scheme in Fig. 1). Our system was designed to automatically calculate the maximum number of parameterizations that can be distributed, according to the available memory on the computing nodes.

## Results

### Recon 2.2, all exchange reactions

As a first test, we applied the SA to the genome-wide metabolic constraint-based model of human metabolism Recon 2.2 [15]. Such model contains 5324 metabolites, 7785 chemical reactions and 1675 genes.

In this test, we investigated the sensitivity of all the $D=693$ exchange fluxes that allow intake of metabolites considered in the original network. For this test, due to memory limitations on the single nodes (i.e., 256 GB) we limited *N* to a value of 2^15^. Thus, we performed an overall number of $N (D + 2) = 22773760$ optimization, distributed across 256 cores in 16 nodes, that is, 88960 parameterizations for each core and 1423360 parameterization on each computing node.

**Fig. 2:**
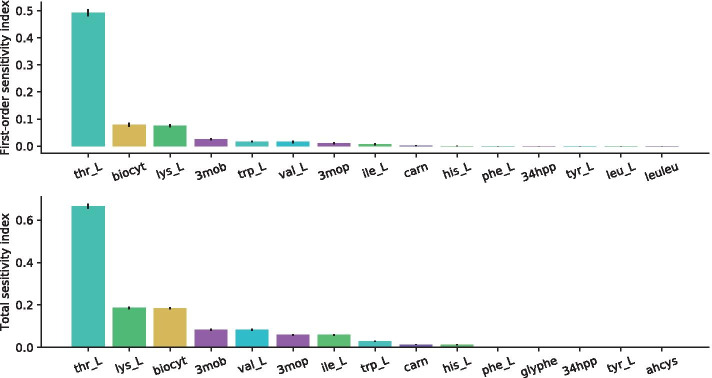
Results of the SA on the Recon2.2 model. First-order sensitivity indices (top) and the total effect indices (bottom) and their 95% confidence level. Ranking 1 to 30 is reported

Figure 2 reports the first order (top panel) and total (bottom) sensitivity coefficients, and their 95% confidence level, of the top 30 ranked parameters.

According to our results, the input variable with the highest first-order sensitivity index is the flux of threonine (Thr_L), an essential amino-acid (see Fig. 2, on top). The second and third in the ranking are biocytin and lysine, respectively. The former is a vitamin, while the latter is an essential amino-acid. The fourth ranked parameter corresponds to the flux of *α*-ketoisovaleric acid (denoted by 3mob in Fig. 2), which is a abnormal metabolite arising as a result of the incomplete degradation of branched-chain amino-acids (leucine, isoleucine and valine).

Tryptophan and valine rank respectively as fifth and sixth most sensitive input, with similar coefficients. Both are essential amino acids (eAAs) and are thus crucial for protein biosynthesis. Besides being a protein component, tryptophan takes part in numerous metabolic reactions, with particular regard to serotonin and nicotinic acid; whereas valine can be utilized as a carbon source to derive energy. In seventh position is another abnormal metabolite: 3-methyl-2-oxovaleric, which is analogous to the previously mentioned one in terms of origin and features. In eight position, isoleucine, which is another essential aminoacid. From ninth position on sensitivity coefficients become negligile.

Switching to the analysis of total sensitivity indices (see Fig. 2, bottom), it can be observed that there is a good consistency with first-order coefficients, at least for the first five raking positions. From fifth position on, the most sensitive metabolites in decreasing order are: (5) valine; (6) 3-methyl-2-oxovaleric; (7) isoleucine; (8) tryptophan (9) isoleucine; (10) carnosine; (11) histidine.

When dealing with total sensitivity, the index associated with valine thus overcomes that associated with tryptophan. Carnosine is dipeptide obtained from the condensation reaction between beta-alanine and L-histidine. It is typically abundant in muscle and brain tissues. Histidine is an essential amino acid.

From twelfth position on, sensitivity indices become negligible.

To draw some conclusion, the most important uptake fluxes, according to total SA, are related to essential amino acids. If, on the one hand, this result could reasonably be expected, on the other hand it is quite surprising that important nutrients such as glucose, glutamine and oxygen display negligible sensitivity indices.

### Recon 2.2, selected reactions

To investigate whether the strongly dominant effect of the most sensitive parameters may hide the importance of less sensitive ones, we performed a new SA on Recon 2.2, by restricting the overall analysis to a subset of the input variables, which is expected to influence biomass production. The selected subset coincides with the intake fluxes modeled in the ENGRO1 network [16], a model of central carbon metabolism, taking into account the main nutrients that are known to play a role in cancer metabolic reprogramming and growth.

Specifically, we restricted the SA to the fluxes describing the intake of glutamine (gin), glucose (glc), oxygen (o_2_), arginine (arg), methionine (met), and tetrahydrofolic acid (thf). In this test, the settings were $D = 6$ and $N = 2^{19}$, leading to a total of 4194304 independent FBA runs.

**Fig. 3:**
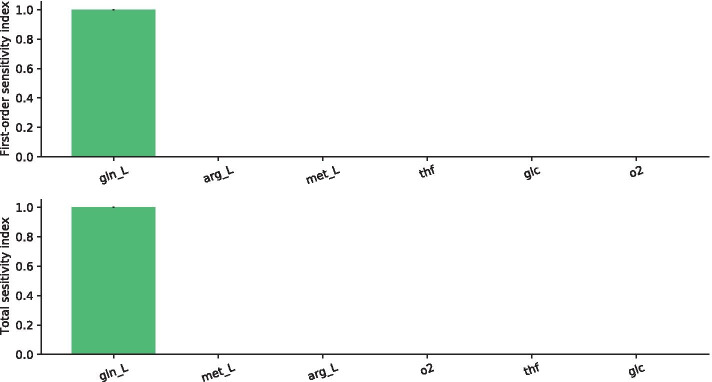
Results of the SA on the Recon2.2 model restricting the analysis to the intake of glutamine (gln), glucose (glc), oxygen (o_2_), arginine (arg), methionine (met), and tetrahydrofolic acid (thf). First-order sensitivity indices (top) and the total effect indices (bottom) and their 95% confidence level are reported

The first order and total sensitivity indices of this restricted SA are shown in Fig. 3. These results clearly indicate that, although the sensitivity indices are not necessarily negligible, surprisingly, glutamine intake is the only sensitive flux, among the six analysed parameters.

### Recon 3D, all reactions

We incremented the scale of the problem by testing the our methodology on the Recon3D model [3], the biggest and most detailed constraint-based metabolic model of human cell to date. This model is composed of 10600 reactions, involving 5835 metabolites and 2248 genes. In this test, we investigated the sensitivity of $D = 1559$ exchange fluxes. Due to memory constraints on the supercomputer that we exploited, the largest setting for *N* was 2^13^, leading to a total of 12787712 FBA runs, distributed over 256 cores in 16 computing nodes (i.e., 49952 runs per core, 799232 runs per node).

**Fig. 4:**
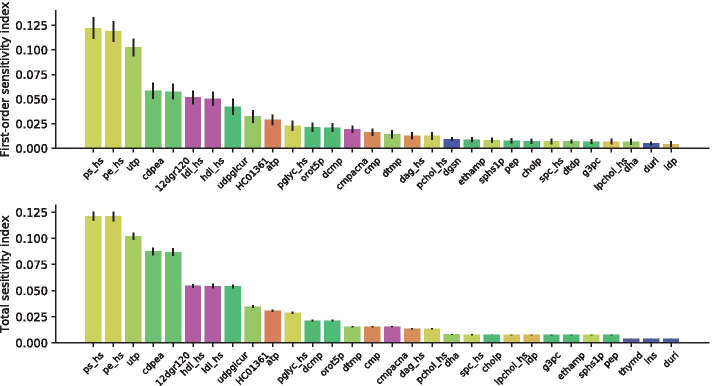
Results of the SA on the Recon 3D model. First-order sensitivity indices (top) and total effect indices (bottom), along with their 95% confidence level. Ranking 1 to 30 is reported

Figure 4 reports the 30 highest-ranked fluxes, in the case of first-order (top) and total effects (bottom) sensitivity indices. The highest-ranked indices—both in the case of first-order and total effects—are related to the exchange fluxes of the following metabolites, in decreasing order of importance: phosphatidylserine (ps_hs), phosphatidylethanolamine (pe_hs), uridine triphosphate (utp), CDP-ethanolamine (cdpea), 1,2-diacyl-*sn*-glycerol (12dgr120), low-density lipoprotein (LDL_HS) and high-density lipoprotein (HDL_HS), commonly known as “bad” and “good” cholesterol, Uridine diphosphate glucuronic acid (udpglcur), 7,8-Dihydroneopterin (HC01361) and adenosine triphosphate (ATP).

Worth of note, as opposed to the case of Recon2.2, the reported most sensitive metabolites do not include essential amino acids. Although the set of most sensitive parameters tends to include lipids, it is more heterogeneous as compared to the Recon2.2 case. To make some examples, the phosphatidylserine and phosphatidylethanolamine are phospholipids that compose plasma membrane or cholesterol, whereas Uridine-5’-triphosphate (UTP) is a pyrimidine nucleoside triphosphate mainly involved in RNA biosynthesis.

Sensitivity indices do not become negligible as quickly as in the Recon2.2 model case, but start to vanish only after the 23rd ranking position. The coefficients for metabolites from 30th to 60th position are reported in Additional file 1: Fig. S1.

### Recon3D, selected reactions

For the reason mentioned in previous section, also for Recon3D model, we performed a SA analysis limited to the input parameters corresponding to the intake fluxes in the ENGRO 1 model [16]. The number of perturbed parameters in the SA is a D = 7, one more than in the Recon2.2 case, because we considered both L and R structure for arginine. In this case we choose to set *N* to 2^18^ samples leading to 12787712 FBA runs.

**Fig. 5:**
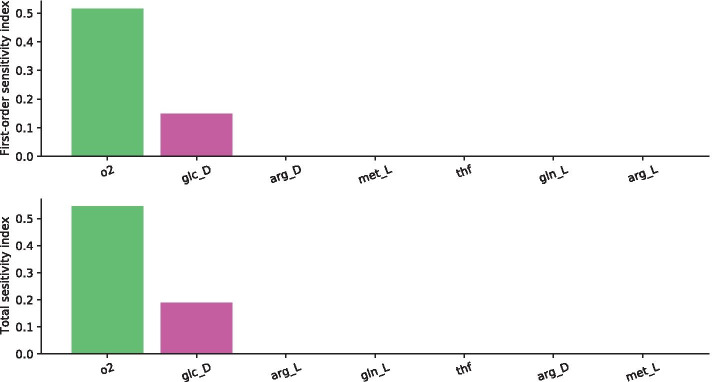
Results of the SA on the Recon3D model restricting the analysis to the intake of glutamine (gln), glucose (glc), oxygen (o_2_), arginine (arg), methionine (met), and tetrahydrofolic acid (thf). First-order sensitivity indices (top) and the total effect indices (bottom) and their 95% confidence level are reported

The sensitivity coefficients reported in Fig. 5 reveal that, consistently with results in [11], oxygen is the most sensitive nutrient, whereas glucose has a relevant but secondary role. Remarkably, in opposition with both results in [11] and with SA results on Recon2.2, glutamine plays a negligible role.

### Parallel acceleration

The CPU time required to execute the SAs on all exchange reactions reported in the previous sections was about 31 days for Recon2 and 38 days for Recon3D. In order to assess the contribution of high performance computing in reasonable time, we performed a further series of less intensive tests (smaller *N*) on D.A.V.I.D.E.. Specifically, we determined the reduction of the computation time for the distributed SA of the Recon 2.2 model using an increasing number of computing nodes. For this test, we used $N=4400$ parameterizations to calculate the sensitivity indices; we calculated the overall running time in the case of distributed calculation over 1, 2, 4, 16 and 32 nodes. Each node is equipped with two POWER8 CPUs, each containing 8 physical cores. We specify that the computation time of FBA simulations is not affected by the specific value of the parameters.

**Fig. 6:**
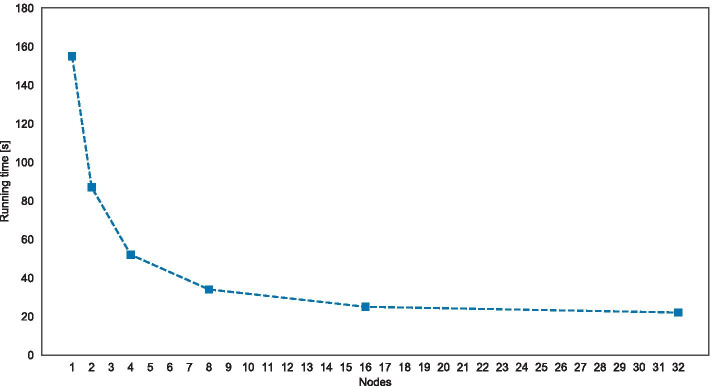
Speedup comparison. Comparison of the running time for the SA of the Recon2.2 model using an increasing number of nodes. The speedup using 32 nodes is approximately $7\times$ with respect to case of a single node

The results in Fig. 6 show that the performance of our method scales linearly up to 16 nodes: the running time is reduced from 155 s (1 node) down to 25 s (16 nodes); after that threshold, the overhead due to communications and data processing (including the competition for intra-node shared resources) exceeds the computation time: by using 32 nodes, the running time is only reduced to 22 s. Hence, the highest speedup that we experienced for this test was equal to $7 \times$; we wish to point out that the speedup could be even higher in the case of larger values of *N*.

Scripts to reproduce results are available at https://github.com/BIMIB-DISCo/Accelerated-globalSA-of-constraint-based-models

## Discussion

To the best of our knowledge, this is the first time a global sensitivity analysis is performed on constraint-based genome-wide models. These models have traditionally been investigated by means of local techniques that compute the derivative of model outputs with respect to the input parameters. Local techniques are computationally efficient but have the drawback of not accounting for interactions between variables and of being related to a fixed nominal point in the space of parameters. For example, in a recent work [28] constraints on all intake fluxes were estimated experimentally and the sensitivity of internal metabolic fluxes was assessed by calculating the percentage change in maximum growth rate over the percentage change in a given flux. Alternatively, the lethality of single reaction deletion has been analyzed with the purpose of assessing critical differences between conditions [29]. Single reaction deletion analysis can be regarded as a naive local sensitivity analysis.

On the contrary, variance-based global SA methods account for interactions between variables and do not depend on the choice of a nominal point, as they assess the effect of an input while all other inputs are varied as well. These methods compute first and higher order coefficients quantifying the importance of different subsets of factors to the output variance, as well as total sensitivity coefficients - accounting for the total contribution to uncertainty due to factor $X_i$ (i.e its first-order effect plus all higher-order effects due to interactions).

Elementary effect (Morris method [21]) and derivative-based global methods [23, 30] are a hybrid class of methods that vary one factor at a time (OAT) alike local methods but are global in the sense that they explore the full parameter space. These methods allow to approximate the total sensitivity index [31], but do not estimate first order (main effect) and higher order coefficients. For this reason, they can be successfully used for identifying non important factors, but not to rank important variables.

**Fig. 7:**
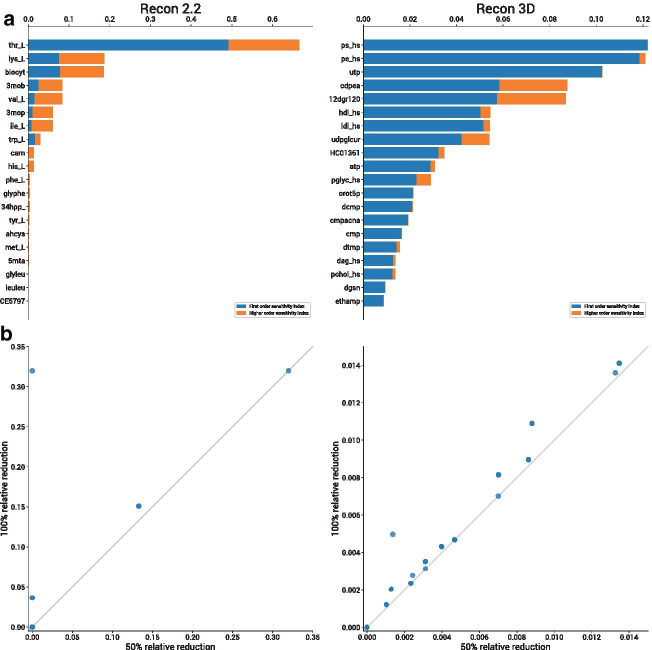
Comparison between Sobol and OAT methods **a** Stacked bar plot of first order coefficient $S_i$ (blue) and difference between total and first order $S_{T_i} - S_i$ (orange) for the the first 20 most influential parameters, for Recon2.2 (left) and Recon3D (right). Possible negative differences due to numerical instability problems are not shown. **b** Scatter plots between the relative reduction of optimal biomass obtained following a 50% reduction and a total depletion of metabolite influxes. The grey line indicates the bisector

Results in Figs. 2, 3, 4 and 5 do not highlight the contribution of higher order effects on the total sensitivity of input factors, as the two indices are intrinsically correlated, given that the total index includes the first order one. To better investigate whether interaction and non linear effects exist in genome-wide metabolic models, Fig. 7 illustrates the difference $S_{T_i}-S_i$ between total and first order coefficient for the first 20 most influential inputs. If this difference is negligible, it means that the dependence of the output *Y* on the input factor $X_i$ is nearly linear, [30]. This is the case for example of phosphatidylserine (ps_hs) and utp in Recon3D (Fig. 7a). However in most cases there is a significant contribution of higher order effects. In particular, the amino acid valine (val_L) and the related metabolite 3mob of in Recon2, display a greater contribution of higher order effects than of first order one. In Recon3D the phenomena is less prominent, still some metabolites, such as cdpea and 12dgr120, show a significant contribution of higher order effects.

To further investigate the issue, we performed a small test of comparison between the results of Morris [21] and of Sobol method applied on Recon 2.2. The Morris method is based on the construction of a series of trajectories in the space of the inputs, where inputs are randomly moved One-At-a-Time (OAT). It estimates the main effect of a factor by computing $\mu *$ , which is defined as the estimate of the mean of the distribution of the absolute values of a number of local measures (the elementary effects) [31] in different regions of the parameter space and the standard deviation ($\sigma$) of the elementary effects. $\mu *$ assesses the overall influence of the factor on the output. The standard deviation $\sigma$ estimates the ensemble of the factor’s effects, whether nonlinear and/or due to interactions with other factors. In Additional file 2: Fig. S2 it can be observed that $\sigma$ is always larger than $\mu *$, implying that the elementary effects relative of each factor differ substantially from one another, indicating that the value of an elementary effect is strongly affected by the choice of the other factors’ values (i.e. of the sample point at which it is computed).

It is worth discussing briefly run-time differences between Morris and Sobol methods. Both analyses were executed with the same (small) number of parametrizations ($N=2^6$) with the same number of logical cores ($n=7$) on a CPU Intel Core i7-9750H (12M Cache, up to 4.50 GHz Clock, 6 Cores). The analysis took a very similar time for both methods (268 s for Morris and 287 s for Sobol); however, as it can be observed in Additional file 2: Fig. S2, the confidence intervals are smaller in the former case. The Morris method is indeed expected to reduce the computational cost, by reducing the number of samples required to narrow the confidence interval [32]. We remind that the 95% confidence intervals have been obtained by bootstrap resampling as in [26], without the need for further model evaluations.

As an additional analysis to evaluate the non linearity of input-output relationship, we performed single perturbations of the admitted flux of the exchange fluxes of the two models and computed the relative reduction in growth rate (optimal biomass reduction over input flux reduction). We investigated the relation between the relative reduction obtained when a total depletion of the flux (100% perturbation) and when a 50% perturbation is simulated. If the sensitivity index was region invariant, we should observe that all values in the scatter plot in Fig. 7 lay on the bisector, but this is not always the case. For example, the effect of a perturbation in L-tryptophan (trp_L) in Recon2, which corresponds to the point that lays far above the bistector line in Fig. 7b, strongly depends on the region in the parameter space. It should be noticed that the effect of nutrient perturbations in Recon3D is generally extremely low. The complete results of the single reaction perturbation analysis are reported in Additional file 3: Fig. S3. The results of this analysis further support the concept that a global strategy is advised when exploring the sensitivity of inputs parameters in genome-wide models.

## Conclusion

We have proposed here a global SA pipeline that produces a sensitivity ranking of growth nutrients in genome-wide constraint-based models, within a few hours, thanks to the use of advanced super-computing infrastructures. To show an application of the pipeline we performed the SA on genome-wide models of human metabolism Recon2.2 and Recon3D. To generate different parametrizations, we systematically and simultaneously perturbed the input variables represented by the boundaries of the exchange fluxes, which are known to greatly influence FBA outcomes [33]. Such parametrizations were distributed to independent FBA optimizations, performed on several processing units according to MPI standard. Finally from the results of the different optimizations, we computed and ranked first order and total sensitivity coefficients.

The obtained ranking largely differs for the two models Recon2.2 and Recon3D. In the former model, most sensitive parameters are mainly associated with the intake of essential amino acids, whereas in the latter they are associated largely with phosholipids but may also relate with nucleotide synthesis. This discrepancy must originate from the differences in the two network reconstructions (whose investigation is however beyond the scope of this work) and suggests that the high sensitivity of essential amino acids intake boundaries in Recon2.2 is not simply a byproduct of their essentiality for biomass production, and thus for positive value of the objective function, but more likely relates with carbon and/or nitrogen metabolism, otherwise high sensitivity would be observed also in Recon3D. However, Fig. 7 shows that both SA coefficients and effects of single reaction deletion analysis tend to display lower values in Recon3D as compared to Recon2.2. Apparently, in Recon3D no reaction is essential for growth: at most a deletion results in a 30% reduction (data not shown). This result is quite surprising and would deserve further investigation, which is however beyond the scope of this work. For both models, we observed a skewed distribution of the values of the sensitivity indices, which cast a shade on the differences between the influence level of less important parameters. Taken together, our results show that our approach can efficiently reveal important differences about the behavior of different genome-wide models.

More importantly, accelerated global SA allows to identify which parameters require additional research for strengthening the knowledge base, thereby reducing output uncertainty. Given the enormous amount of different nutrients used by a cell, especially in vivo, it is indeed of paramount relevance to restrict the number of related parameters that must be measured. The divergences between first order and total order coefficients that we observed indicate that interactions phenomena between perturbations in nutrient intake fluxes are not always negligible, thus local SA methods such as single reaction deletion analyses should be applied with caution to genome-wide models. When a parameter has interactions with other ones, to properly set the value of that parameter, all the interacting parameters must also be set to their correct value. Hence, one should explore the Sobol higher order coefficients for that parameter, which can also be returned by our approach, to identify the metabolites it interferes with.

Along similar lines, SA may suggest which parameters are insignificant, providing indications for model reduction.

Finally, once one is reasonably confident about model constraints, SA explains which nutrients most highly correlate with growth rate or other metabolic functions.

From a computational point of view, the SA could be accelerated further in future versions of the algorithm, by dedicating an entire node, rather than a single core, to the sampling of the parameter space, in order to free memory that could be allocated to increase the number of tested parametrizations, thus further reducing 95% confidence intervals.

## Supplementary Information

**Additional file 1: Fig. S1.** Results of the SA on the Recon3D. First-order sensitivity indices (top) and the total effect indices (bottom) and their 95% confidence level. Ranking 31 to 60 is reported.

**Additional file 2: Fig. S2.** Comparison between Sobol and Morris coefficients for Recon2.2 model (*N* = 2^6^). A) Total sensitivitycoefficients (μ^*^) obtained with Morris method (top) and scatterplot of μ^*^ vs standard deviation *σ* of elementary effects (bottom). B) First order (top) and total (bottom) sensitivitycoefficients obtained with Sobol methods.

**Additional file 3: Fig. S3.** Reaction deletion analysis. A) Percentageof reduction of the optimal growth rate for a 100% (top plot) or 50% (bottom plot) reduction of maximum allowed flux for eachexchange reaction of Recon 2.2 model. B) Same as A for Recon3D model.

## Data Availability

Not applicable.

## Abbreviations

Ahcys: S-Adenosylhomocysteine
Arg: Arginine
Atp: Adenosine triphosphate
Biocyt: Biocytin
Carn_L: Carnosine
CBM: Constraint-Based Model
Cdpea: CDP-ethanolamine
Cholp: Phosphorylcholine
Cmp: Cytidine monophosphate
Cmpacna: Cytidine monophosphate N-acetylneuraminic acid
Dag_hs: Diglyceride
Dcmp: Deoxycytidine monophosphate
Dgsn: Deoxyguanosine
Dha: Dihydroxyacetone
Dtdp: Thymidine-5′-diphosphate
Dtmp: Thymidine-5′-phosphate
Duri: Deoxyuridine
Ethamp: O-Phosphoethanolamine
FBA: Flux Balance Analysis
eAA: Essential amino acid
Glc: Glucose
Gln: Glutamine
Glyphe: Glycyl-Phenylalanine
G3pc: Glycerophosphocholine
HC01361: 7,8-Dihydroneopterin
Hdl_hs: High-density lipoprotein
His_L: L-Histidine
Idp: Inosine 5′-(trihydrogen diphosphate)
Ile_L: L-Isoleucine
Ins_L: Inosine
Ldl_hs: Low-density lipoprotein
Leu_L: L-Leucine
Leuleu: Leucylleucine
Lpchol_hs: 1-Acyl-Sn-Glycero-3-Phosphocholine
Lys_L: L-Lysine
MPI: Message Passing Interface
Met: Methionine
OAT: One-at-a-time
Orot5p: Orotidylic acid
O_2_: Oxygen
Pchol_hs: Phosphatidylcholine
Pe_hs: Phosphatidylethanolamine
Pep: Phosphoenolpyruvic acid
Pglyc_hs: Phosphatidylglycerol
Phe_L: L-Phenylalanine
Ps_hs: Phosphatidylserine
SA: Sensitivity Analysis
Spc_hs: LysoSM(d18:1)
Sphs1p: Sphingosine 1-phosphate
Thf: Tetrahydro-folic acid
Thr_L: Threonine
Trp_L: L-Tryptophan
Thymd: Thymidine
Tyr_L: L-Tyrosine
Udpglcur: Uridine diphosphate glucuronic acid
Utp: Uridine triphosphate
Val_L: Valine
12dgr120: 1,2-diacyl-sn-glycerol
smob: *α*-ketoisovaleric acid
34hpp: 4-Hydroxyphenylpyruvic acid
