# Supplementary figures and images for: Accelerated global sensitivity analysis of genome-wide constraint-based metabolic models
Source: BMC Bioinformatics. 2021 Apr 26;22(Suppl 2):78. doi: 10.1186/s12859-021-04002-0 (PMC8074438; doi:10.1186/s12859-021-04002-0)

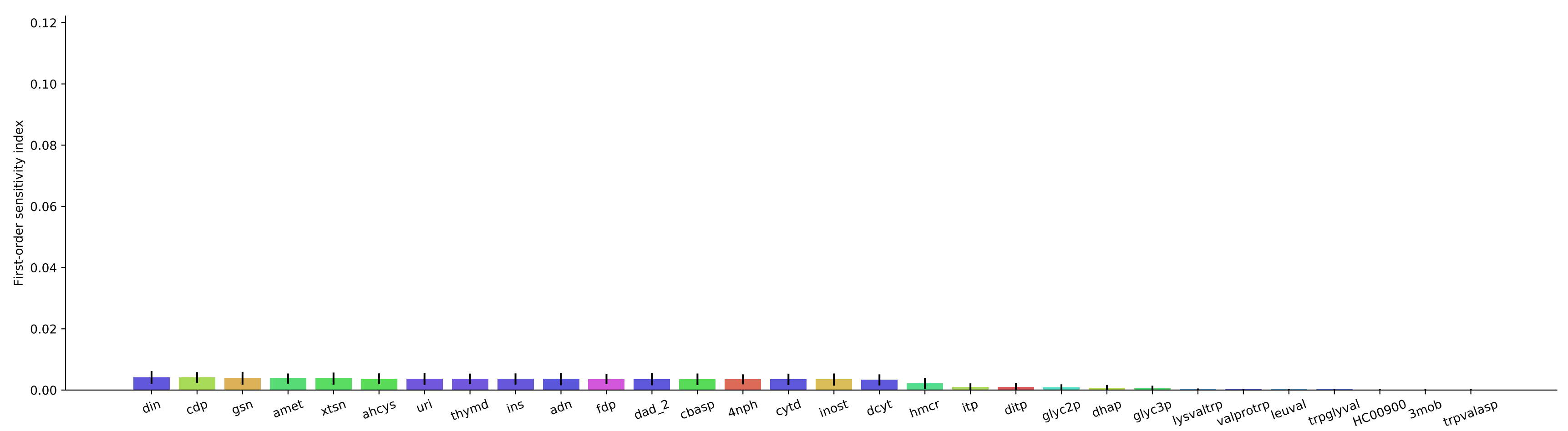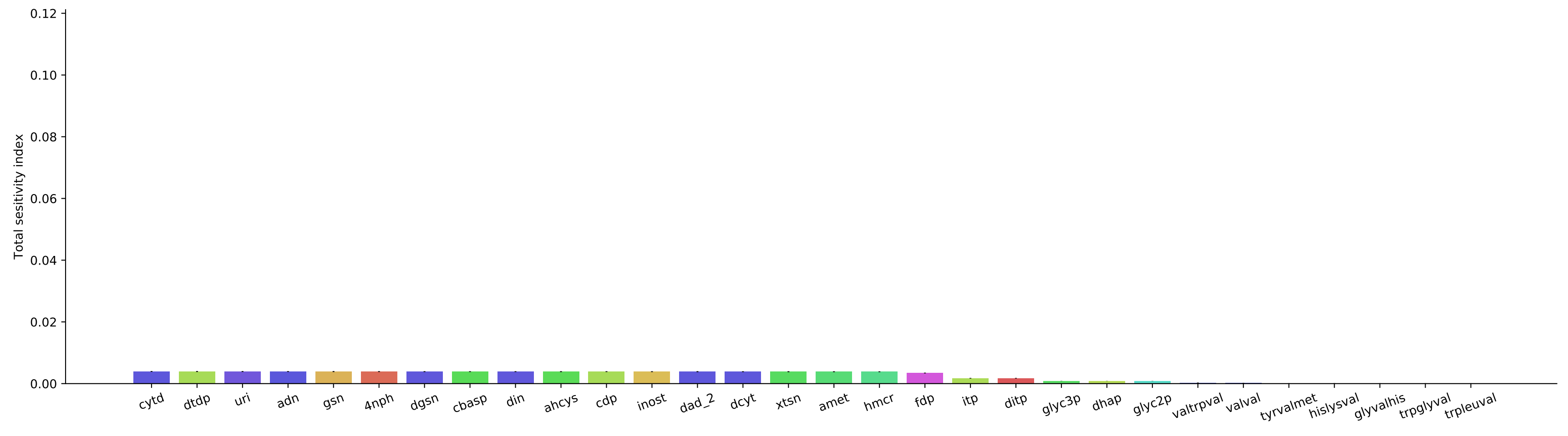

Supplement: Supplementary file 1 — Additional file 1: Fig. S1. Results of the SA on the Recon3D. First-order sensitivity indices (top) and the total effect indices (bottom) and their 95% confidence level. Ranking 31 to 60 is reported. [file 12859_2021_4002_MOESM1_ESM.pdf]

A

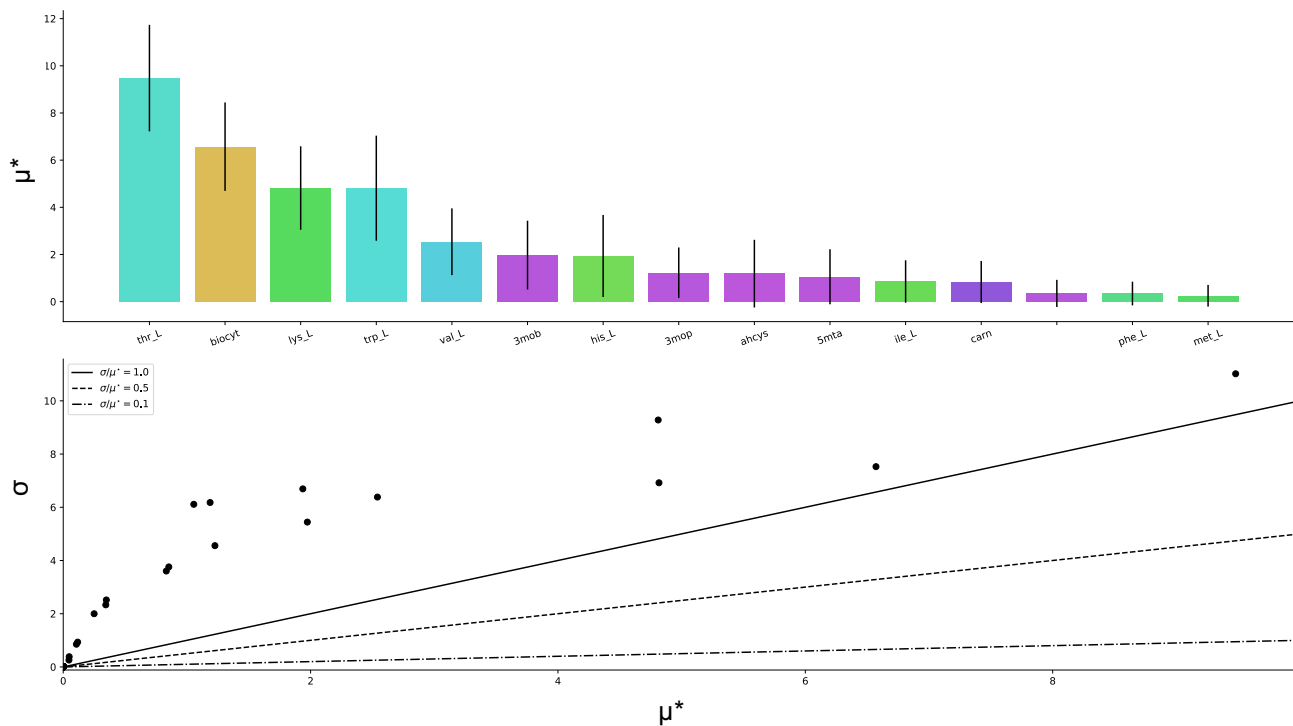

B

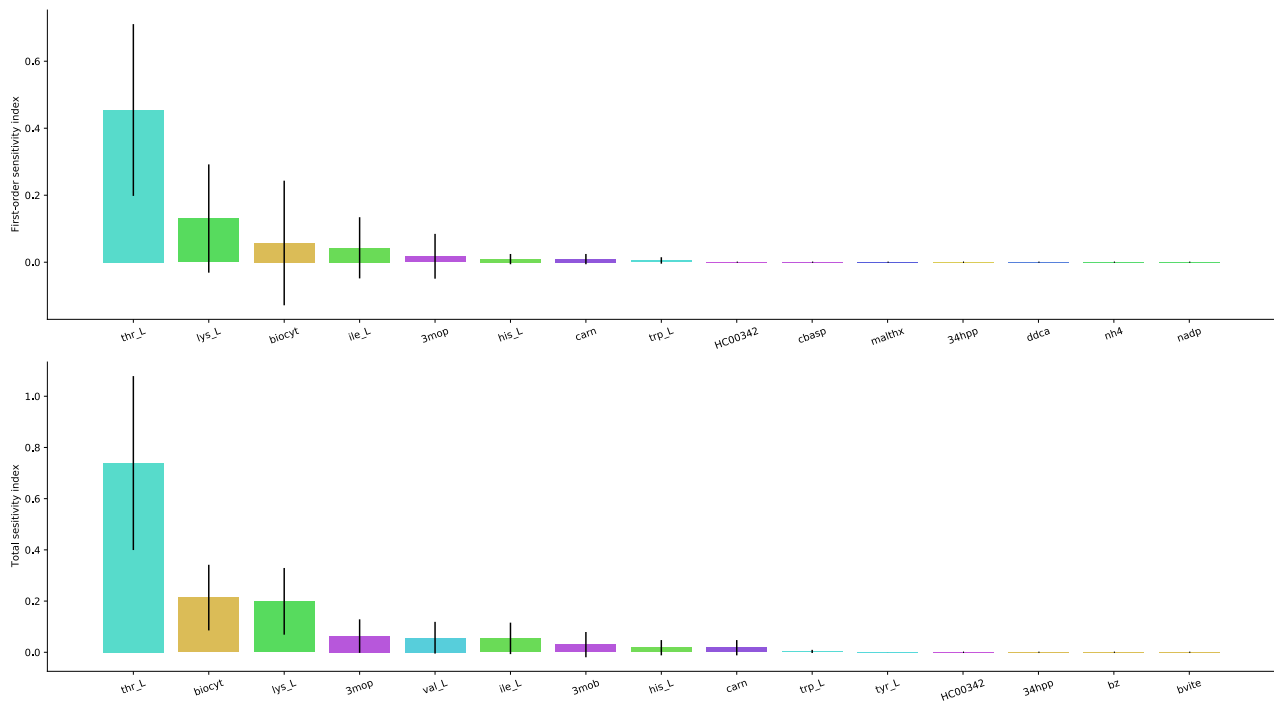

Supplement: Supplementary file 2 — Additional file 2: Fig. S2. Comparison between Sobol and Morris coefficients for Recon2.2 model (N = 26). A) Total sensitivitycoefficients (μ*) obtained with Morris method (top) and scatterplot of μ* vs standard deviation σ of elementary effects (bottom). B) First order (top) and total (bottom) sensitivitycoefficients obtained with Sobol methods. [file 12859_2021_4002_MOESM2_ESM.pdf]

## Recon 2.2

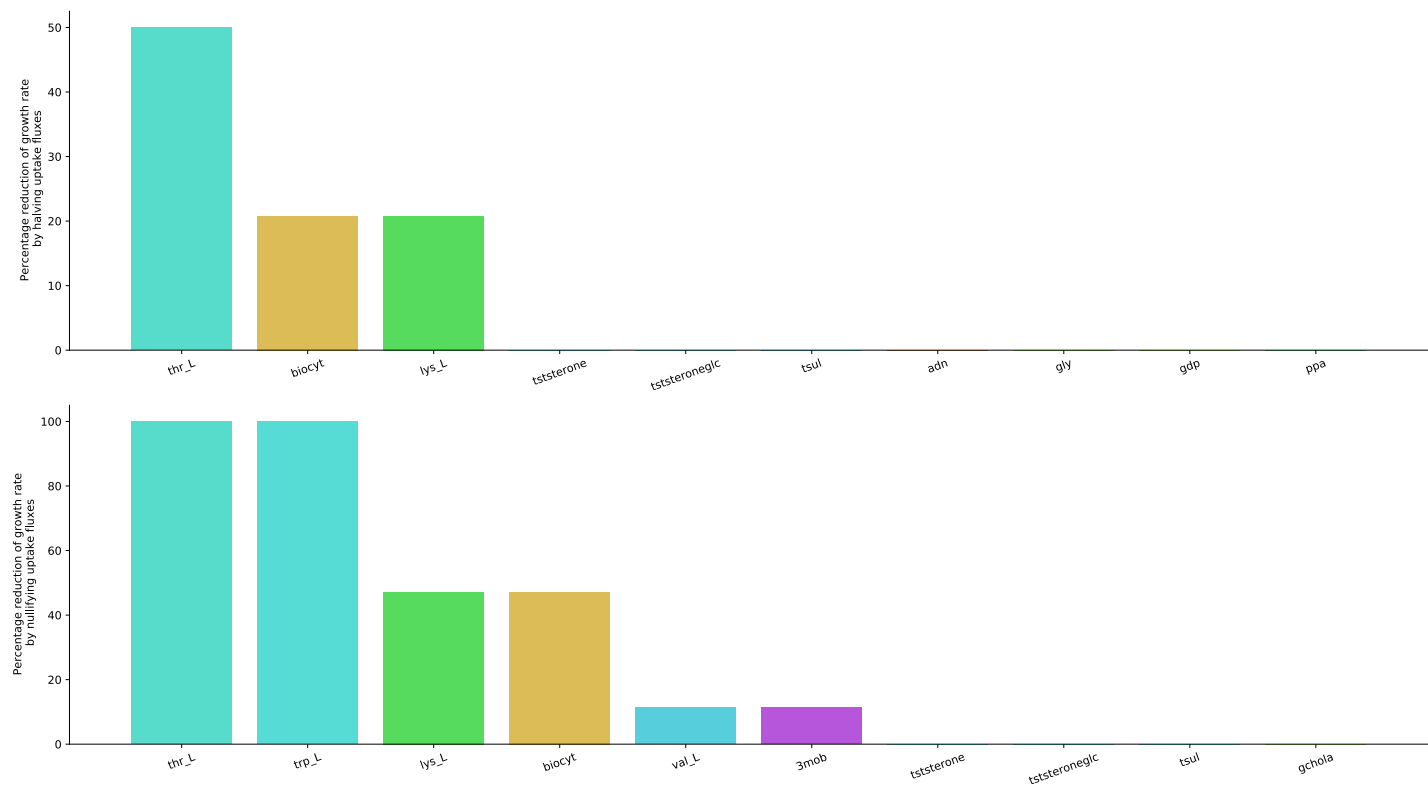

B Recon 3D

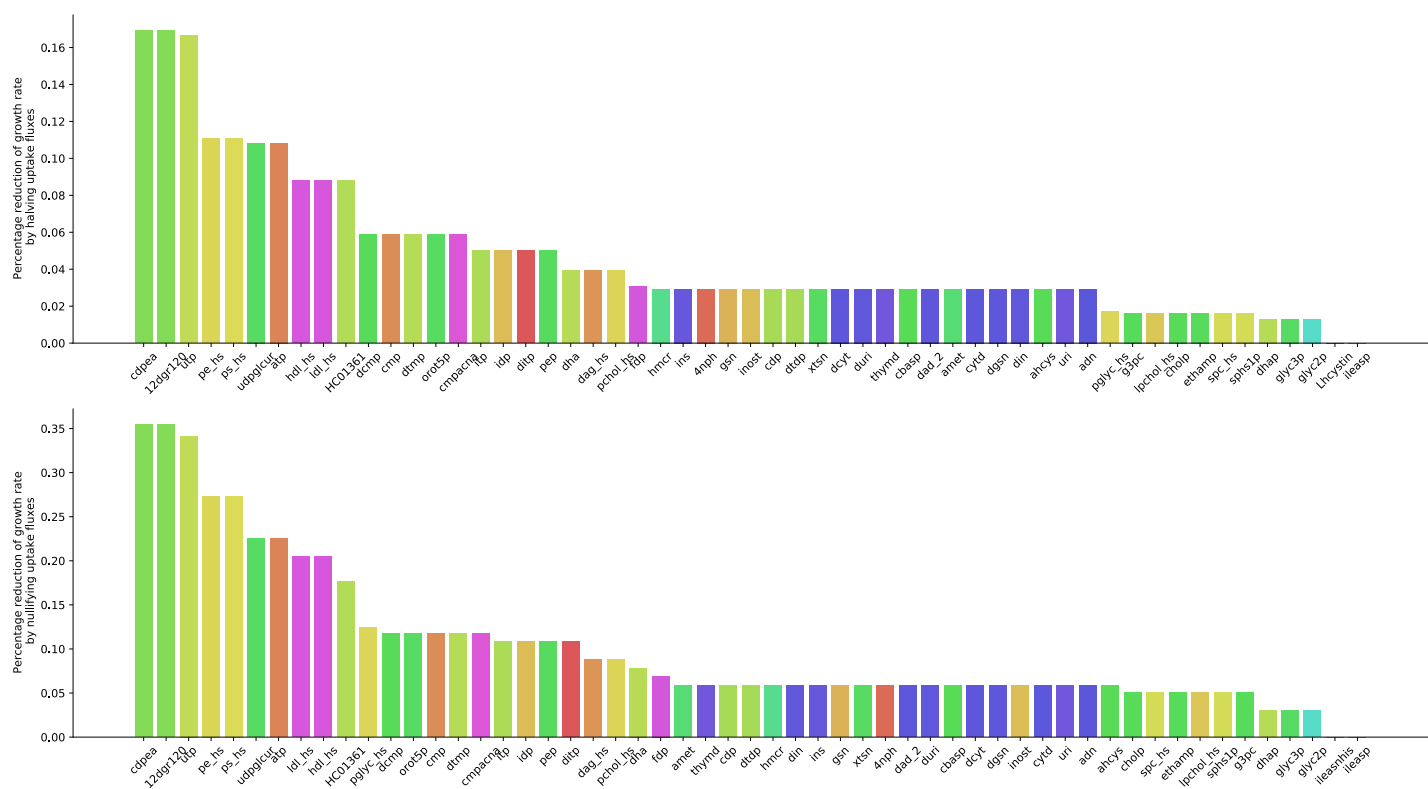

Supplement: Supplementary file 3 — Additional file 3: Fig. S3. Reaction deletion analysis. A) Percentageof reduction of the optimal growth rate for a 100% (top plot) or 50% (bottom plot) reduction of maximum allowed flux for eachexchange reaction of Recon 2.2 model. B) Same as A for Recon3D model. [file 12859_2021_4002_MOESM3_ESM.pdf]
